# Identifying Common Genes, Cell Types and Brain Regions Between Diseases of the Nervous System

**DOI:** 10.3389/fgene.2019.01202

**Published:** 2019-11-29

**Authors:** Mengling Qi, Shichao Fan, Zhi Wang, Xiaoxing Yang, Zicong Xie, Ken Chen, Lei Zhang, Tao Lin, Wei Liu, Xinlei Lin, Yan Yan, Yuedong Yang, Huiying Zhao

**Affiliations:** ^1^Sun Yat-sen Memorial Hospital, Guangzhou, China; ^2^Guangdong Provincial Key Laboratory of Malignant Tumor Epigenetics and Gene Regulation, Guangzhou, China; ^3^School of Data and Computer Science, Sun Yat-sen University, Guangzhou, China; ^4^Software Institute, Nanjing University, Nanjing, China; ^5^Department of Hepatobiliary Surgery II, Zhujiang Hospital of Southern Medical University, Guangzhou, China; ^6^Zhongshan Medical College, Sun Yat-sen University, Guangzhou, China; ^7^School of Public Health, Sun Yat-sen University, Guangzhou, China

**Keywords:** diseases of the nervous system, genetic similarity of diseases, disease-related genes, phenotypic similarity of diseases, disease similarity in cell types, disease similarity in brain region

## Abstract

**Background:** Diseases of the nervous system are widely considered to be caused by genetic mutations, and they have been shown to share pathogenic genes. Discovering the shared mechanisms of these diseases is useful for designing common treatments.

**Method:** In this study, by reviewing 518 articles published after 2007 on 20 diseases of the nervous system, we compiled data on 1607 mutations occurring in 365 genes, totals that are 1.9 and 3.2 times larger than those collected in the Clinvar database, respectively. A combination with the Clinvar data gives 2434 pathogenic mutations and 424 genes. Using this information, we measured the genetic similarities between the diseases according to the number of genes causing two diseases simultaneously. Further detection was carried out on the similarity between diseases in terms of cell types. Disease-related cell types were defined as those with disease-related gene enrichment among the marker genes of cells, as ascertained by analyzing single-cell sequencing data. Enrichment profiles of the disease-related genes over 25 cell types were constructed. The disease similarity in terms of cell types was obtained by calculating the distances between the enrichment profiles of these genes. The same strategy was applied to measure the disease similarity in terms of brain regions by analyzing the gene expression data from 10 brain regions.

**Results:** The disease similarity was first measured in terms of genes. The result indicated that the proportions of overlapped genes between diseases were significantly correlated to the DMN scores (phenotypic similarity), with a Pearson correlation coefficient of 0.40 and *P-value* = 6.0×10^-3^. The disease similarity analysis for cell types identified that the distances between enrichment profiles of the disease-related genes were negatively correlated to the DMN scores, with Spearman correlation coefficient = -0.26 (*P-value* = 1.5 × 10^-2^). However, the brain region enrichment profile distances of the disease-related genes were not significantly correlated with the DMN score. Besides the similarity of diseases, this study identified novel relationships between diseases and cell types.

**Conclusion:** We manually constructed the most comprehensive dataset to date for genes with mutations related to 20 nervous system diseases. By using this dataset, the similarities between diseases in terms of genes and cell types were found to be significantly correlated to their phenotypic similarity. However, the disease similarities in terms of brain regions were not significantly correlated with the phenotypic similarities. Thus, the phenotypic similarity between the diseases is more likely to be caused by dysfunctions of the same genes or the same types of neurons rather than the same brain regions. The data are collected into the database NeurodisM, which is available at http://biomed-ai.org/neurodism.

## Background

The nervous system helps all parts of the body to effectively communicate with each other, and diseases of the nervous system usually cause problems in the central and peripheral neurons. One of the important features of these diseases is that they often occur simultaneously in the same person or with similar symptoms that are considered to be caused by common mechanisms (Kohli et al., 2013; Boison and Aronica, 2015).

The common mechanisms of diseases include the same gene or mutation causing multiple diseases. For instance, one mutation in the *TBK1* gene has been shown in both Amyotrophic Lateral Sclerosis (ALS) and Frontotemporal Dementia (FTD) patients (Le Ber et al., 2013; Borghero et al., 2016), and frameshift and truncating mutations (such as exon 8 IVS+3G→A and exon 6 549 + 1delG) in the *PRKAR1A* gene cause Carney complex due to nonsense-mediated mRNA decay (Kirschner et al., 2000; Sun et al., 2015), whereas another mutation in the exon 11 (c.1101C→T) was found to cause acrodysostosis by truncating the last 14 amino acids in the coded cAMP-binding protein domain B protein due to the introduction of a stop codon (Linglart et al., 2011). The shared mutations or genes between diseases can potentially assist the discovery of new pathogenic mutations or genes (Zhao et al., 2017).

The most important step toward identifying the shared mutations or genes between diseases is to collect data reflecting the relationship between mutations and the diseases. With the development of sequencing technology, many disease-causing genes have been discovered (Zhu et al., 2017) and a few databases have been constructed to present the relationship between genes and phenotypes (Fredman et al., 2002; Stenson et al., 2017). For example, the ALPL gene mutations database was designed for hypophosphatasia (Antonarakis, 1998; den Dunnen and Antonarakis, 2003); another database including 614 mutations in 14 genes was specifically designed for Alzheimer’s disease, Frontotemporal dementia, and Parkinson’s disease (Cruts et al., 2012); NeuroDNet is a database collecting 300 genes related to 12 neurodegenerative diseases (Vasaikar et al., 2013); PDmutDB is a database collecting all known mutations and non-pathogenic coding variations in genes related to Parkinson’s disease, which includes 192 pathogenic mutations and 231 mutations with unclear functions (Nuytemans et al., 2010; Cruts et al., 2012); EpilepsyGene (http://61.152.91.49/EpilepsyGene) is a database containing mutations and genes related to Epilepsy (Ran et al., 2015).

The increasing number of disease-related genes in the databases is now making identification of the common mechanisms of diseases in cell types and brain regions more possible. A recent study was performed to discover the widely expressed brain regions and cell types of the genes related to epilepsy (Delahaye-Duriez et al., 2016). However, no similar study has been performed for diseases of the nervous systems. Nevertheless, many mutations related to diseases of the nervous system are being uncovered by increasing numbers of genomic investigations, which provides us the opportunity to detect the shared mechanisms of the diseases in terms of genes, cell types, and brain regions (Hoischen et al., 2014; Van Cauwenberghe et al., 2016).

In this study, we focused on 20 heritable diseases of the nervous system to discover their shared genes, cell types, and brain regions. These 20 diseases were selected because they were included in the International Classification of Diseases for mortality and morbidity statistics (10th Revision) and were collected in the Online Mendelian Inheritance in Man (OMIM) database (Hamosh et al., 2005) (https://www.omim.org/) as heritable diseases. To obtain the genes related to these diseases, we manually reviewed 518 English-language articles published between 2007 to 2017 in PubMed and compiled a list of 1607 mutations in 365 genes identified by sequencing technology. To make the dataset comprehensive, we combined these with 837 pathogenic mutations in 114 genes from the Clinvar database (Landrum et al., 2014). The genetic similarities of the diseases were measured by the number of genes shared between them. The relationship between the genetic similarity and the phenotypic similarity was evaluated by the phenotype similarity scores from the Disease Manifestation Network (DMN). The DMN scores of these disease pairs were found to be significantly correlated with the proportion of genes shared between diseases. The genes related to the diseases were further analysed to identify which types of neurons they were enriched in. The gene enrichment profiles were constructed by plotting the enrichment of these genes in 25 cell types. The disease similarity in the cell types was then obtained by calculating the distances between enrichment profiles. The results indicated that the enrichment profile distances were significantly correlated with the phenotypic similarity scores. The same strategy was applied to identify the relationship between the disease-related genes and 10 brain regions. However, no significant correlation was identified between the distances between the brain region enrichment profiles and the phenotypic similarity scores, while the diseases with lower similarity in terms of brain regions tended to have lower phenotypic similarity. Another interesting result of this study was that migraine-related genes were found to be enriched in radial glia-like cells. This is the first time that a relationship between migraine and radial glia-like cells has been reported.

## Materials and Methods

### Data Collection

We compiled a list of mutations related to diseases of the nervous system through searching the literature. The diseases included in this study total 20 heritable diseases defined by the OMIM database (https://omim.org/). Briefly, the names of the nervous system diseases were obtained from the World Health Organization (2016) International Classification of Diseases for mortality and morbidity statistics (10th Revision) (ICD10) (https://icd.who.int/browse10/2016/en). In the ICD10, we clicked the disease cluster "Disease of the nervous system" and accessed the first layer of disease categories related to diseases of the nervous system. From these categories, a total of 99 classifications of diseases were obtained, which were used as standard disease names to query the Online Mendelian Inheritance in Man (OMIM) database (Hamosh et al., 2005). Out of 99 diseases, 20 diseases were included in the OMIM database as heritable diseases, and these were selected for this study. These diseases are Alzheimer’s disease (AD), amyotrophic lateral sclerosis (ALS), aphasia, basal ganglia disorder (BG), cerebral palsy (CP), dystonia, epilepsy, frontotemporal dementia (FTD), ataxia, hydrocephalus, migraine, multiple sclerosis (MS), muscular atrophy (MA), muscular dystrophy, myopathies, paraplegia, Parkinson’s disease (PD), polyneuropathy, stroke, and tetraplegia.

We searched the PubMed database by using MeSH terms including disease names for articles published between January 2007 and September 2017. This led to 518 useful publications, from which we manually curated all phenotypic and genotypic data. The mutations included in this study had been identified as disease-related by next-generation sequencing technology, and their functional impacts in genes had been experimentally validated. We downloaded the Clinvar database (Landrum et al., 2014) (July 2018) (https://www.ncbi.nlm.nih.gov/clinvar/) and extracted the "pathogenic" or "likely pathogenic" mutations related to diseases of the nervous system.

### Annotation of Mutations

All mutations were converted to hg19 through LifOver (Hinrichs et al., 2006) and were annotated for functional category by ANNOVAR (Wang et al., 2010a).

### Cell-Type Analysis

A publicly available set of cell-type marker genes was used to pinpoint whether genes are specifically enriched in a particular cell type (La Manno et al., 2016). The single-cell RNA-seq data were obtained from ventral midbrain in humans and included 25 cell subtypes belonging to 13 cell types (La Manno et al., 2016) (**Table S1**). For a set of genes, we aimed to identify which cell types they were enriched in. The enrichment of genes in a certain cell type was evaluated according to the numbers of nervous system disease genes overlapped with the cell-type marker genes. The enrichment was evaluated by Fisher exact test (FET, two-tail). If the P-values were less than 0.05, the cell type was considered to be enriched with nervous system disease genes.

### Gene Expression Analysis in Different Human Brain Regions

The gene expression dataset (GEO accession number: GSE60862) includes gene expression data for ten brain regions: cerebellar cortex, frontal cortex, occipital cortex, temporal cortex, hippocampus, putamen, thalamus, medulla, white matter, and substantia nigra. The data were obtained by measurements in 1,231 European-descent individuals collected by the UK Brain Expression Consortium (UKBEC) (Trabzuni et al., 2013). The gene expression data from different brain regions were analyzed with the limma package (Smyth, 2004). A gene was defined to be highly expressed in a brain region if its expression was at least one time higher than the average expression levels in other regions and the difference is significant with a P-value < 0.05. A gene subset was defined as significantly enriched in the brain region if the number of highly expressed genes was significantly higher than expected by chance according to the Fisher’s exact test.

### Genetic Similarity of Diseases

We evaluated the genetic similarity between diseases on the basis of the number of shared genes through the Chi-square test, following the same method as in our previous work (Zhao et al., 2017).

### Similarity of Diseases in Terms of Cell Types and Brain Regions

The similarities of diseases in terms of cell types and brain regions were calculated by comparing the enrichment of the genes in 25 types of cells. The enrichment differences were measured as the distances between the enrichment score profiles of two diseases. The enrichment score was calculated by Equation 1. A profile of the enrichment score for one disease was obtained from the enrichment scores of the genes related to the disease in 25 types of neurons. The profile of enrichment score in the different brain regions included 10 factors that were obtained from the enrichment scores of the genes related to the disease in 10 brain regions. The profile distances were calculated by the R package, "dist" using the "binary" method, which regards the values in the profiles as binary.

(1) $$\text{Enrichment score}=\frac{\text{NOverG}}{NG_{1}\times NG_{2}}$$

where *NOverG* denotes the number of overlapped genes of two gene sets; *NG* *_1_* and *NG* *_2_* represent the number of genes included in two gene sets, respectively.

### Other Databases Involved in This Study

The Disease Manifestation Network (DMN) is a database-creating phenotype network based on the usage of highly accurate disease-manifestation semantic relationships from the Unified Medical Language System (UMLS) (Chen et al., 2015). DMN provides similarity scores between a pair of diseases, with a higher score representing a higher similarity between one pair of phenotypes. If one pair of diseases have multiple matching, the average value of the DMN scores is used.

The Clinvar database (http://www.ncbi.nlm.nih.gov/clinvar/) is a freely accessible, public archive report of the relationships among human variations and phenotypes (Landrum et al., 2014). As of January 2018, the database collected a total of 314,218 variants.

## Results

### Data Collection

By manually reviewing 518 articles, we collected 1,607 pathogenic mutations in 365 genes that are related to 20 diseases of the nervous system occurring in 3,779 samples from 83 countries. In comparison, only 837 pathogenic mutations in 114 genes related to the 20 diseases were collected in the Clinvar database, totals that are 1.9 and 3.2 times lower than those in our collection. Only ten mutations and 54 genes were common to our collection and the Clinvar database. A combination of the two datasets led to a total of 2434 mutations and 424 genes. Among these, 86 genes were shared by at least two diseases, and the remaining 338 genes were related to only one disease.

### Evaluating Genetic Similarity Between Diseases

According to the sharing of genes with disease-causing mutations, we measured genetic similarities between 20 diseases. In total, 46 out of 190 pairs were discovered to share significant numbers of genes (P-value < 0.05) according to the Chi-square test. Meanwhile, when evaluating the genetic similarity of diseases by the sharing of mutations, 21 disease pairs were identified with P-value < 0.05. The greater number of significant pairs based on genes than based on mutations is consistent with our previous observation because different mutations on one gene might cause the same functional change (Zhao et al., 2017). **Table S2** lists the genetically similar disease pairs evaluated by different methods.

In comparison, the use of the Clinvar database only identified four disease pairs as sharing significant numbers of genes. For the disease pairs sharing mutations, only one disease pair was identified by Clinvar.

### Comparing Genetic Similarity Between Diseases With Phenotypic Similarity

**Figure 1:**
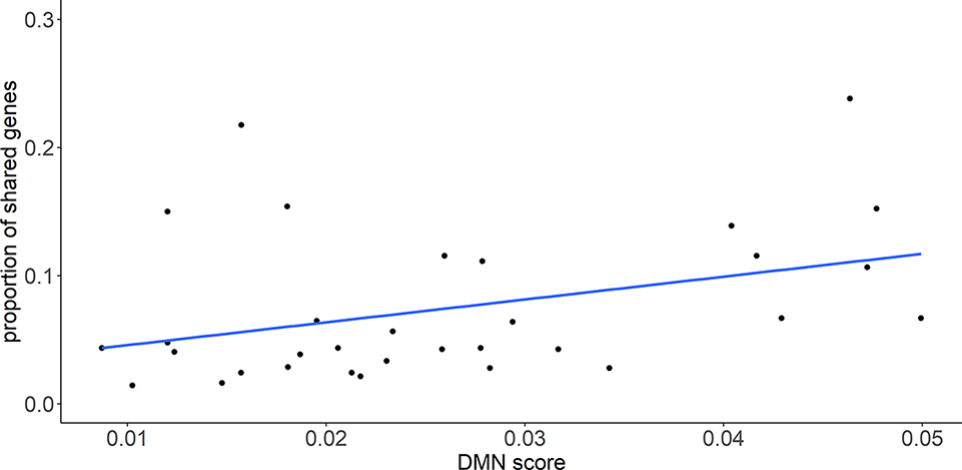
The proportion of shared genes between diseases is significantly correlated with the phenotypic similarity score (DMN scores). Pearson correlation coefficient (PCC) is 0.40 with P-value = 6.0 × 10^-3^.

To evaluate the relationship between the genetic similarity and phenotypic similarity of diseases, we mapped disease pairs to the DMN database. The average value of the DMN score was used to represent the phenotypic similarity of a disease pair because the DMN database provides multiple scores for each disease pair. The genetic similarity was also measured by the proportion of shared genes, which was defined as the number of overlapped genes divided by the smaller number of disease-related genes in the pair of diseases. In total, 39 disease pairs shared genes and had a phenotypic similarity score in the DMN database. The proportion of shared genes was found to be significantly correlated with the DMN score, with a Pearson correlation coefficient (PCC) and a P-value of 0.40 and 1.2×10^-2^, respectively (**Figure 1**). This result potentially reflects the relationship between genetic similarity and phenotypic similarity.

### Enrichment of Disease-Related Genes in Cells

**Figure 2:**
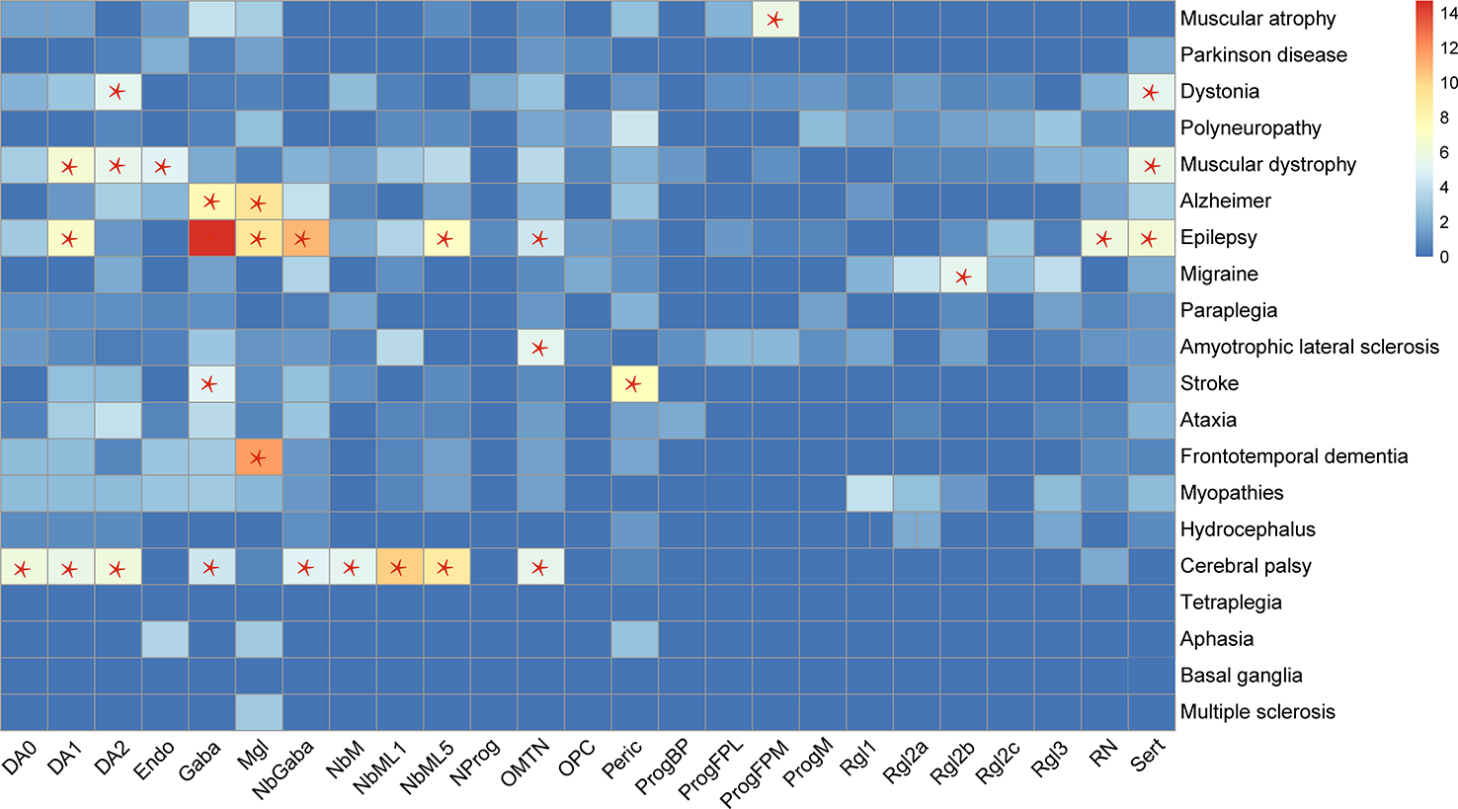
Cell types enriched by genes related to specific diseases. Red star, "*", denotes a significantly enriched neuron type. The enrichment of 365 genes in 25 cell types was analyzed in this study. The enrichment of the genes in the cell types was evaluated by Fisher exact test (FET, two-tail), which evaluated the number of genes used as cell marker genes. If the FET P-value was less than 0.05, the cell type was considered as enriched with nervous system disease genes.

By analyzing the cell types enriched in the genes related to certain diseases, 15 types of cells were found to be enriched in the genes related to nine kinds of diseases (**Figure 2**). Most of the relationships between the diseases and cell types are well studied. One novel finding is on the relationship between the migraine and radial glia-like cells, which has not been reported before.

Another interesting finding is on cerebral palsy and epilepsy. The cell types enriched by genes related to cerebral palsy cover the highest number of the cell types enriched by epilepsy-related genes (**Figure 2**), which may reflect the similarity between these two diseases. The well-known similarity between them is that they occur commonly in children and affect development. However, the cerebral palsy genes are not enriched in Microglia, which were enriched in the genes related to epilepsy, frontotemporal dementia, and Alzheimer’s disease (AD). A recent single-cell study in AD has identified significantly upregulated genes in oligodendrocytes, astrocytes, and microglia of AD patients (Mathys et al., 2019), which is consistent with our finding on the relationship between microglia and AD.

### Evaluating Disease Similarity in Cell Types

**Figure 3:**
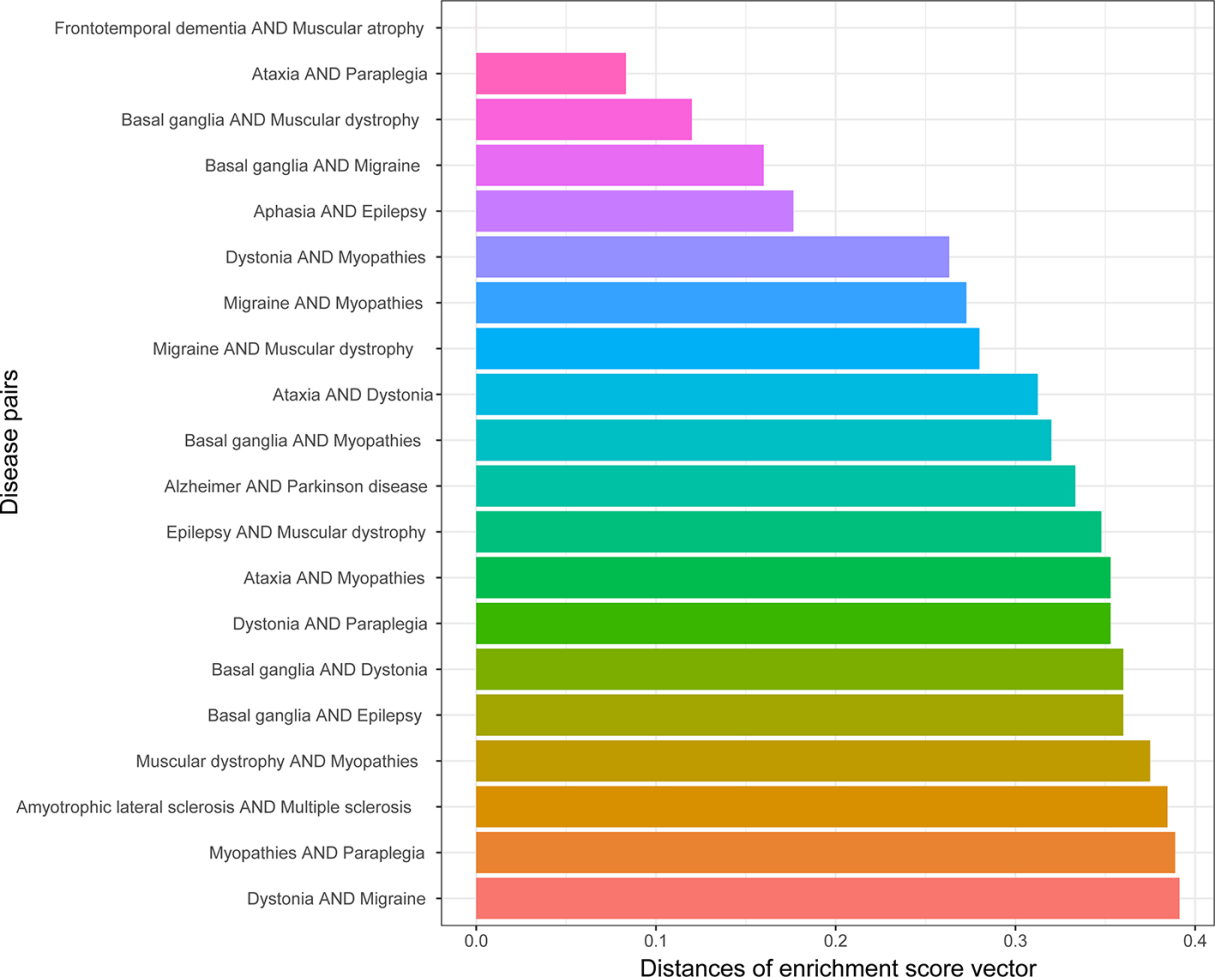
Top 20 disease pairs with the lowest distances between their cell type enrichment profiles.

We obtained the enrichment score profile on 25 types of neurons for each gene set related to one disease. The similarity of the diseases in cell types were defined as the profile distances for 190 disease pairs. A smaller distance means that two profiles are more similar. **Figure 3** shows the top 20 most similar disease pairs. Among these disease pairs, Alzheimer’s disease and Parkinson’s disease are the most popular diseases affecting older people, and amyotrophic lateral sclerosis (ALS) and multiple sclerosis (MS) are two diseases with many similar symptoms (Beesley et al., 2018). Meanwhile, five out of the 20 disease pairs were identified as being genetically similar (**Table 1**).

**Table 1:** Disease pairs were identified as genetically similar and were ranked to obtain the top 20 with the lowest distances between cell type enrichment score profiles.

Disease pair	Distance ^a^	P-value ^b^
Frontotemporal dementia/Muscular atrophy	0	2.1 × 10^-114^
Ataxia/Paraplegia	0.88	4.4 × 10^-9^
Alzheimer’s disease/Parkinson’s disease	0.33	4.4 × 10^-59^
Epilepsy/Muscular dystrophy	0.35	1.1 × 10^-11^
Muscular dystrophy/Myopathies	0.38	< 2×10^-16^

^a^Distance between cell type enrichment profiles.

^b^P-values of genetic similarity analysis.

**Figure 4:**
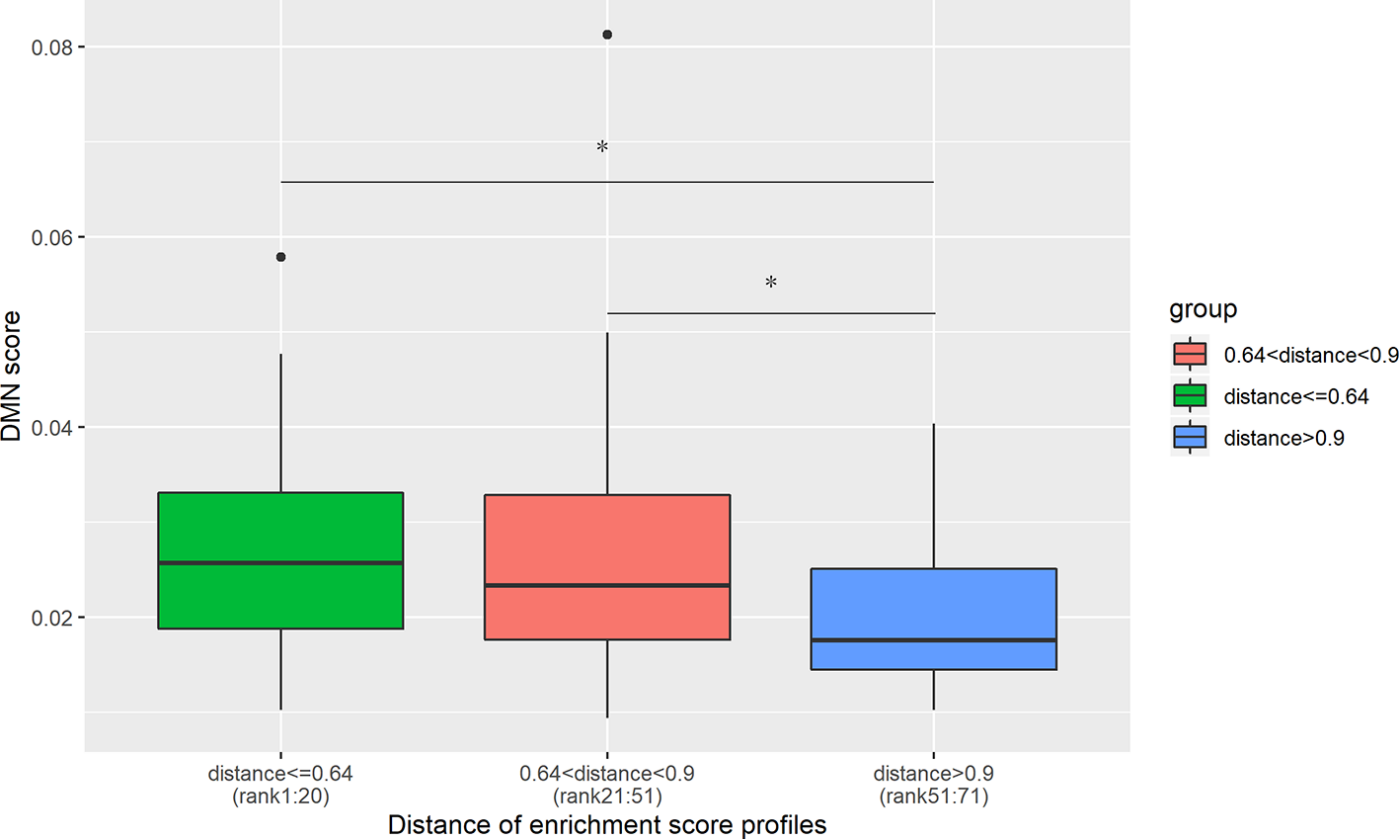
DMN scores decrease with an increase in the cell type enrichment profile distance. The top 20 disease pairs with the lowest enrichment profile distances have significantly higher DMN scores than the 20 disease pairs with the highest enrichment profile distances. The average DMN scores for the three groups of disease pairs are 0.0278, 0.027, and 0.020, respectively. *denotes the t-test significance with P-value < 0.05.

Out of 190 disease pairs, 71 have phenotypic scores in the DMN database. The phenotypic scores were discovered to be significantly correlated with the enrichment profile distances, with Spearman correlation coefficient -0.26 and P-value = 3.1×10^-2^ (**Figure S1**). The disease pairs were ranked from the lowest enrichment profile distance to the highest. The top 20 disease pairs with the lowest distances showed higher DMN scores than the 20 disease pairs with the highest distance (**Figure 4**). Meanwhile, the DMN scores of the disease pairs ranking 21 to 51 are significantly higher than the 20 disease pairs with the highest distances (**Figure 4**).

### Enrichment of Disease-Related Genes in Brain Regions

**Figure 5:**
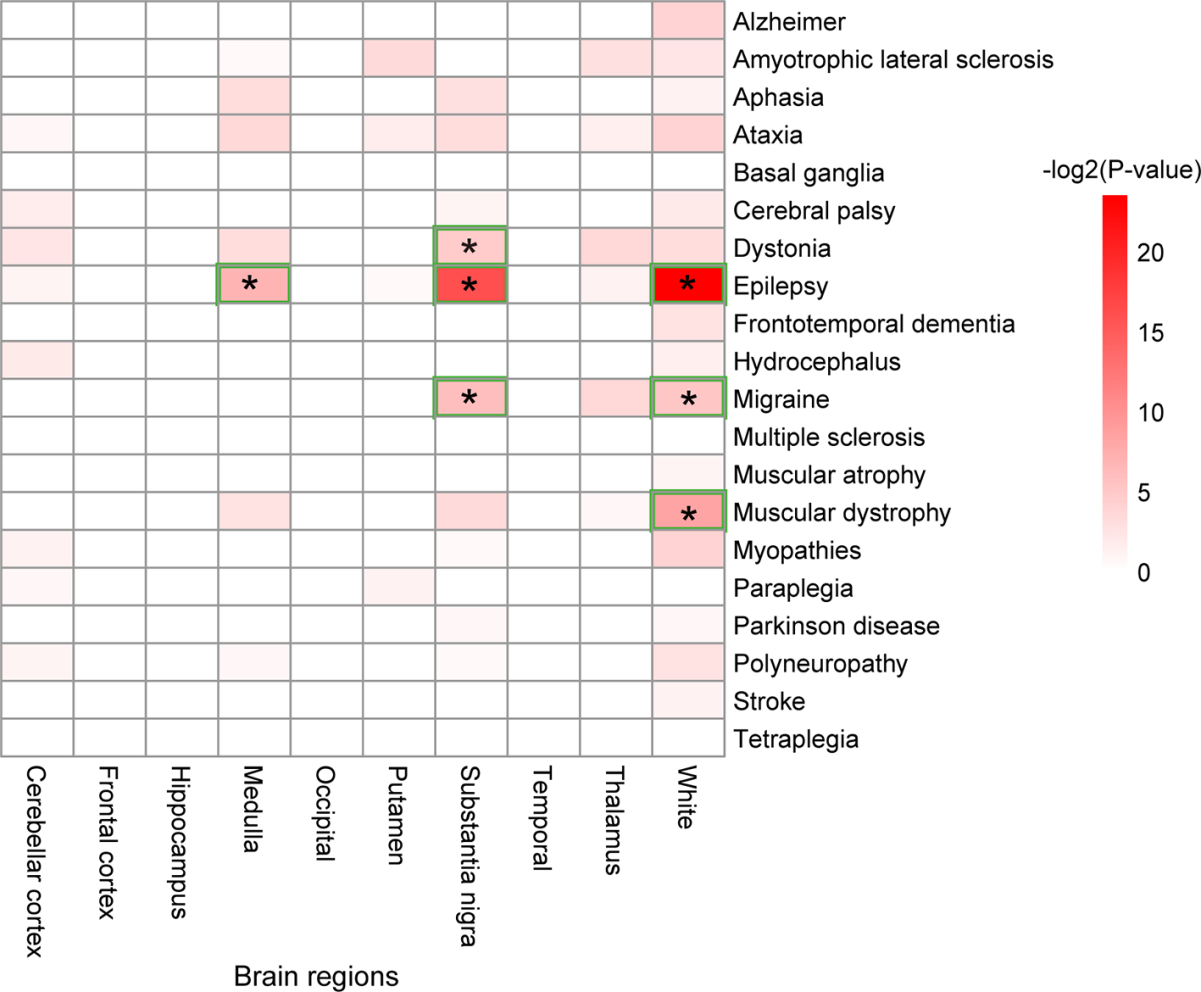
Brain regions enriched by the disease-related genes. A star, "*", denotes a brain region significantly enriched by the genes related to a disease.

Further analysis is carried out to uncover the brain regions in which the genes related to a certain disease play important roles (**Figure 5**). As shown in **Figure 5**, white matter is the brain region enriched by the genes related to epilepsy, migraine, and muscular dystrophy. The relationships between white matter and these three diseases were well studied. A recent study has discovered the relationship between white matter and epilepsy by evaluating the microstructural properties of superficial white matter in regions corresponding to u-fibers (Ostrowski et al., 2019). The relationship between white matter and migraine was evaluated by a study using magnetization transfer imaging (Arkink et al., 2019). For the roles of white matter in muscular dystrophy, researchers have discovered widespread white matter abnormalities in patients through magnetic resonance imaging (MRI) (Mendell et al., 2006).

The substantia nigra brain region is enriched by genes related to three disease types: migraine, epilepsy, and dystonia. The medulla is a brain region that is only enriched by the genes related to epilepsy.

### Evaluating Disease Similarity in Brain Regions

**Figure 6:**
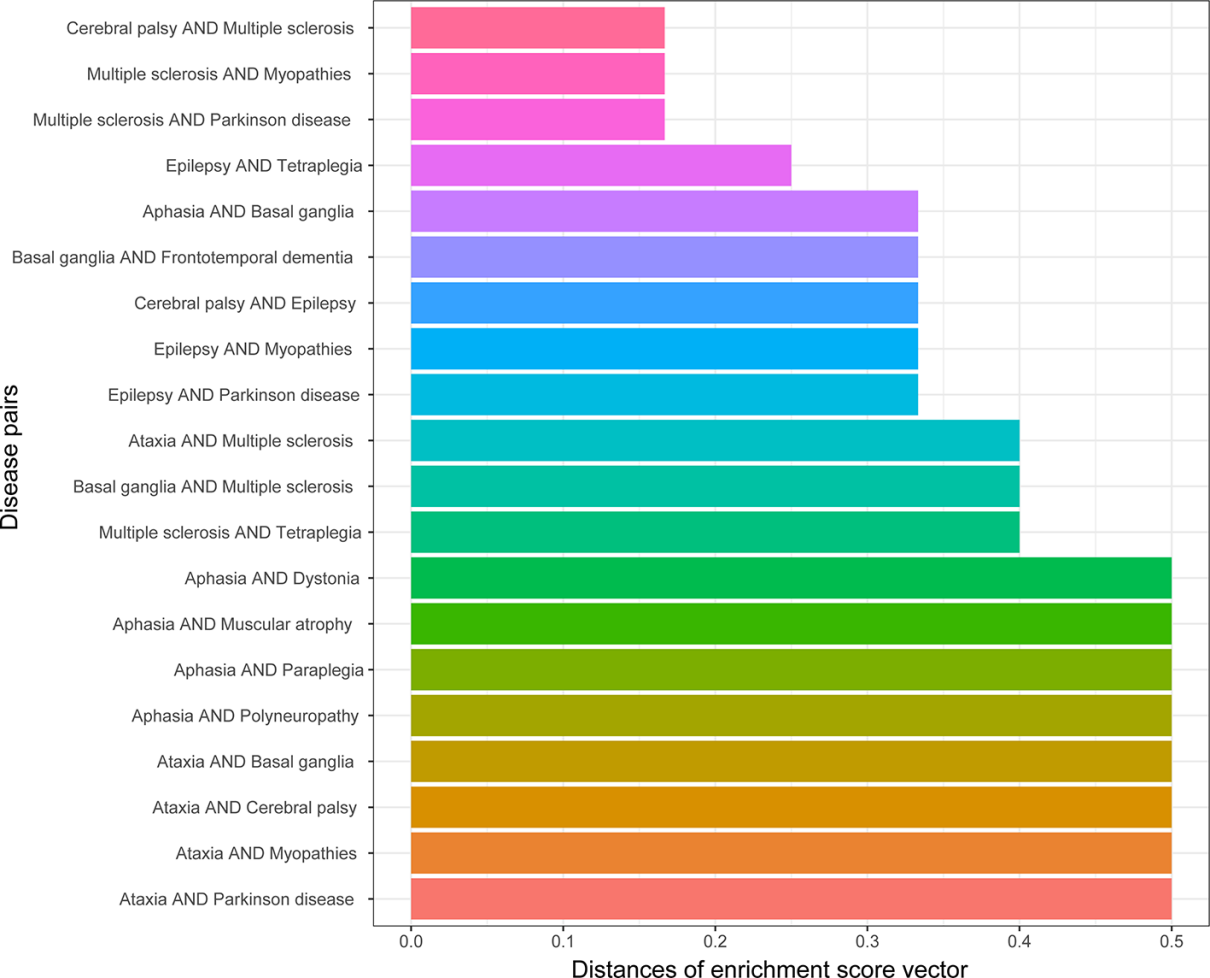
Top 20 disease pairs with the lowest brain region enrichment profile distances.

The similarity of two diseases in terms of brain regions was obtained by calculating the distances between gene enrichment profiles. The lower the distance means two diseases, the more similar they are in terms of brain regions. The top 20 disease pairs with the lowest distance are shown in **Figure 6**. Among them, only one disease pair (ataxia and myopathies) were included in the top 20 most similar diseases in terms of cell types, and one disease pair (epilepsy and Parkinson’s disease) was detected to be genetically similar.

**Figure 7:**
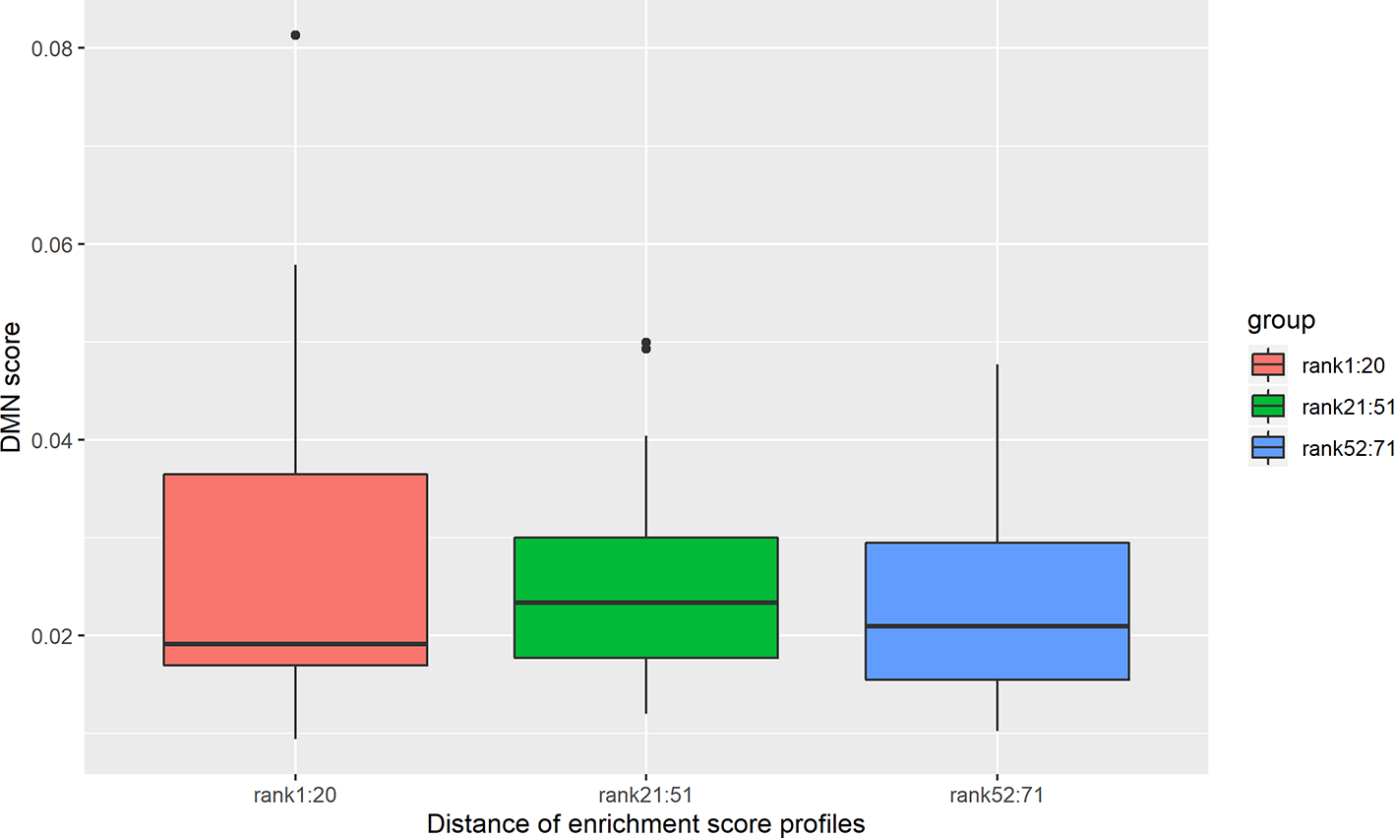
DMN scores decrease with an increase in the brain region enrichment profile distance. The disease pairs were clustered into three groups according to enrichment profile distance. The first group is the top 20 disease pairs with the lowest distances. The second group is the disease pairs ranked from 21 to 51. The third group is the 20 disease pairs with the highest distances. The average DMN scores for the three groups are 0.028, 0.025, and 0.023, respectively.

There are 71 disease pairs with DMN scores. When the distances between their enrichment profiles were compared to the DMN scores, no significant correlation was discovered. Nevertheless, we found that the top 20 disease pairs with the lowest enrichment profile distances have higher DMN scores (average 0.028) than the 20 disease pairs with the highest enrichment profile distances (average DMN score = 0.023). The results are shown in **Figure 7**.

## Discussion

This study aimed to identify the shared genes, cell types, and brain regions of nervous system diseases. By constructing a mutation database for 20 diseases, the genetic similarity between the nervous system diseases was found to be significantly correlated with the phenotypic similarity. Further analysis was performed to detect the similarity of diseases in terms of cell types and brain regions. The disease similarities in terms of cell types were evaluated by the distance between their enrichment profiles. The enrichment profiles represent the enrichment distributions of the disease-related genes in 25 cell types, and the distance between them reflects the similarity of diseases in terms of cell types. A similar strategy was applied to identify the disease similarity in terms of brain regions. From the top 20 most similar disease pairs in terms of cell types, five pairs were identified to be genetically similar, and only one pair was found in the top 20 most similar diseases in terms of brain regions. In comparison, from the top 20 most similar disease pairs in terms of brain regions, only one pair was uncovered to be genetically similar. Thus, diseases similar in terms of cell types were more likely to share a significant number of genes than to be related to genes enriched in the same brain regions.

Interestingly, this study found that the genes related to cerebral palsy and epilepsy were enriched in the highest numbers of cell types (**Figure 2**). Cerebral palsy and epilepsy are two diseases that may be caused by dysfunctions of more than one kind of cell. The neurons causing these two diseases may possibly result in other diseases of the nervous system, simultaneously. However, the genes related to cerebral palsy were only highly expressed in the frontal cortex and temporal brain regions. Thus, cerebral palsy is a disease that is resulted from mutations in genes as markers in a wide range of cell types in two brain regions. In comparison, the genes related to epilepsy were enriched in eight cell types and highly expressed in nine brain regions, which implicated epilepsy as a disease that affects a wide variety of brain regions and neurons.

Furthermore, this study identified that radial glia might play important roles in migraine development. Migraine is a complex genetic disorder with an estimated heritability of approximately 50% and is thought to be caused by an interplay of multiple genetic variants, along with environmental factors. Many studies have attempted to identify the primary cell types influencing migraine. Recently, the International Headache Genetics Consortium aimed to identify the specific cell types and pathways involved in migraine (Eising et al., 2016). They found that migraine-associated genes were enriched in oligodendrocytes and astrocytes. However, that study only investigated five types of cells, neurons, astrocytes, myelinating oligodendrocytes, microglia, and endothelial cells, and did not consider the impact of the radial glia. In fact, the roles of the radial glia in Parkinson’s disease (Singh et al., 2018) and Alzheimer’s disease (Diniz et al., 2019) have been well studied. Although it is suggested that valproate (VPA) can treat migraine by activating the wnt/beta-catenin pathway, which regulates cell regeneration and differentiation, especially glial cell differentiation and regeneration (Wang et al., 2010b; Moon et al., 2018), the specific regulation mechanism is not clear. Thus, further studies are required to explore the relationship between radial glia cells and migraine.

Meanwhile, diseases of the nervous system were found to frequently share 72 genes, which reminds us that these genes may play important roles in causing multiple nervous system diseases. We further compared the functions of the shared genes with the functions of the unshared genes. The results indicated that the shared genes are involved in distal axon-related cellular components and biological progress including synaptic transmission, glutamatergic neurotransmission, and responding to oxidative stress (**Figure S2**).

The shared genes are also specifically enriched in endomembrane system organization, establishment of organelle localization, and inclusion body assembly. These six functions (synaptic transmission, glutamatergic neurotransmission, responding to oxidative stress, endomembrane system organization, establishment of organelle localization, and inclusion body assembly) were found to sinvolve 29 shared genes (*PLA2G6, PSEN1, RELN, STXBP1, ATP1A2, ALS2, TARDBP, CHMP2B, SOD1, VRK1, SYNJ1, REEP1, DCTN1, DNM2, LMNA, DYSF, SQSTM1, DYNC1H1, MAPT, SACS, VCP, DNAJB6, PNKP, PRKRA,GCH1, C19orf12 and SETX*) that were related to 15 nervous system disease (dystonia, Parkinson’s disease, Epilepsy, Alzheimer’s disease, frontotemporal dementia, aphasia, ataxia, migraine, paraplegia, amyotrophic lateral sclerosis, muscular atrophy, polyneuropathy, muscular dystrophy, stroke, myopathies).

In summary, this study provided a comprehensive database, NeurodisM, including mutations and genes related to 20 diseases of the nervous system. Analyzing the genes in the database identified the genetic relationship and the similarities in terms of neurons and brain regions of the diseases, which may provide clues for further study of treatment designs for the diseases. Meanwhile, this study identified a relationship between migraine and radial glia-like cells, a novel insight into the mechanisms of migraine.

## Data Availability Statement

Publicly available datasets were analyzed in this study. This data can be found here: OMIM database (https://omim.org/), Clinvar (http://www.ncbi.nlm.nih.gov/clinvar/),GEO accession number: GSE60862.

## Author Contributions

MQ: Data curation, formal analysis, methodology, writing—original draft. SF and ZW: Data curation, formal analysis, methodology. XY and ZX: Investigation, methodology. KC: Supervision, investigation. LZ: Investigation. TL: Validation. WL: Methodology. XL: Supervision. YaY: Supervision, software. YuY: Validation, project administration. HZ: Conceptualization, writing review and editing, funding acquisition.

## Funding

Contract Grant Sponsor: the National Key R&D Program of China (2018YFC0910500), the Natural Science Foundation of China (81971190, 81801132, U1611261 and 61772566), the Guangdong Frontier & Key Tech Innovation Program (2018B010109006, 2019B020228001), the Introducing Innovative and Entrepreneurial Teams (2016ZT06D211) and Guangdong Province Key Laboratory of Malignant Tumor Epigenetics and Gene Regulation (2017B030314026) programs, and the Guangdong Provincial Key R&D Program (2018B030337001).

## Conflict of Interest

The authors declare that the research was conducted in the absence of any commercial or financial relationships that could be construed as a potential conflict of interest.
