# Supplementary material for: Identifying Common Genes, Cell Types and Brain Regions Between Diseases of the Nervous System
Source: Front Genet. 2019 Nov 29;10:1202. doi: 10.3389/fgene.2019.01202 (PMC6895906; doi:10.3389/fgene.2019.01202)
Supplement: Supplementary file 1 [file DataSheet_1.docx]

**Table S1. T****he 25 subtypes cell types of the human ventral midbrain.**

| **Abbreviation** | **Full name** |
| --- | --- |
| DA0 | dopaminergic neurons |
| DA1 |  |
| DA2 |  |
| Endo | endothelial cells |
| Gaba | GABAergic neurons |
| Mgl | Microglia |
| NbGaba | neuroblast GABAergic neurons |
| NbM | mediolateral neuroblasts |
| NbML1 |  |
| NbML5 |  |
| NProg | neuronal progenitor |
| OMTN | oculomotor and trochlear nucleus |
| OPC | oligodendrocyte precursor cells |
| Peric | Pericytes |
| ProgBP | Prog, progenitor medial floorplate (FPM), lateral floorplate (FPL), midline (M), basal plate (BP) |
| ProgFPL |  |
| ProgFPM |  |
| ProgM |  |
| Rgl1 | radial glia-like cells |
| Rgl2a |  |
| Rgl2b |  |
| Rgl3 |  |
| Rgl2c |  |
| Sert | Serotonergic |
| RN | red nucleus |

**Table S2. The disease pairs showing genetic similarity in different evaluation methods. The third column the evaluated by shared genes between disease pairs while the forth column by shared mutations.**

| Disease 1 | Disease 2 | Gene p^a^ | Mut p^b^ |
| --- | --- | --- | --- |
| muscular atrophy | polyneuropathy | 2.60×10^-120^ | 8.34×10^-37^ |
| muscular atrophy | muscular dystrophy | 7.18×10^-2^ |  |
| muscular atrophy | Alzheimer disease | 3.23×10^-4^ |  |
| muscular atrophy | Epilepsy | 1.79×10^-5^ | 1.96×10^-37^ |
| muscular atrophy | spastic paraplegia | 2.18×10^-2^ | 4.47×10^-1^ |
| muscular atrophy | Amyotrophic lateral sclerosis | 6.15×10^-233^ | 9.02×10^-236^ |
| muscular atrophy | Ataxia | 3.06×10^-3^ |  |
| muscular atrophy | frontotemporal dementia | 2.14×10^-114^ |  |
| Parkinson’s disease | Dystonia | 3.61×10^-110^ | 3.14×10^-36^ |
| Parkinson’s disease | Alzheimer disease | 4.35×10^-59^ | 2.83×10^-7^ |
| Parkinson’s disease | Epilepsy | 1.82×10^-10^ |  |
| Parkinson’s disease | Amyotrophic lateral sclerosis | 1.45×10^-10^ | 2.11×10^-1^ |
| Parkinson’s disease | frontotemporal dementia | 3.24×10^-88^ |  |
| Parkinson’s disease | Aphasia | 5.66×10^-148^ |  |
| Dystonia | muscular dystrophy | 2.32×10^-1^ |  |
| Dystonia | Alzheimer disease | 9.28×10^-3^ |  |
| Dystonia | Epilepsy | 1.73×10^-47^ |  |
| Dystonia | spastic paraplegia | 1.09×10^-7^ |  |
| Dystonia | Ataxia | 3.60×10^-58^ | 1.47×10^-4^ |
| Dystonia | frontotemporal dementia | 1.12×10^-3^ |  |
| Dystonia | Aphasia | 3.13×10^-13^ |  |
| Polyneuropathy | Epilepsy | 3.29×10^-1^ |  |
| Polyneuropathy | spastic paraplegia | 2.03×10^-84^ | 5.99×10^-14^ |
| Polyneuropathy | Amyotrophic lateral sclerosis | 1.45×10^-10^ |  |
| Polyneuropathy | Ataxia | 9.57×10^-16^ | 1.15×10^-3^ |
| Polyneuropathy | frontotemporal dementia | 1.03×10^-4^ |  |
| Polyneuropathy | Myopathies | 4.62×10^-3^ | 2.14×10^-28^ |
| muscular dystrophy | Epilepsy | 1.06×10^-11^ | 2.20×10^-1^ |
| muscular dystrophy | spastic paraplegia | 3.38×10^-1^ |  |
| muscular dystrophy | Amyotrophic lateral sclerosis | 2.37×10^-4^ | 6.47×10^-2^ |
| muscular dystrophy | Stroke | 1.00×10^-2^ | 4.74×10^-2^ |
| muscular dystrophy | Ataxia | 1.71×10^-1^ |  |
| muscular dystrophy | frontotemporal dementia | 2.51×10^-2^ | 3.91×10^-1^ |
| muscular dystrophy | Myopathies | 0 | 0 |
| Alzheimer disease | Amyotrophic lateral sclerosis | 6.96×10^-13^ |  |
| Alzheimer disease | Stroke | 8.23×10^-7^ |  |
| Alzheimer disease | frontotemporal dementia | 0 | 2.18×10^-89^ |
| Alzheimer disease | Aphasia | 7.78×10^-183^ | 2.71×10^-20^ |
| Epilepsy | Migraine | 2.67×10^-52^ | 2.35×10^-5^ |
| Epilepsy | Ataxia | 5.38×10^-76^ | 9.15×10^-3^ |
| Epilepsy | cerebral palsy | 1.35×10^-1^ |  |
| Epilepsy | Aphasia | 1.21×10^-4^ | 6.40×10^-42^ |
| Migraine | Ataxia | 1.38×10^-77^ | 2.18×10^-90^ |
| spastic paraplegia | Amyotrophic lateral sclerosis | 2.27×10^-15^ | 6.63×10^-1^ |
| spastic paraplegia | Ataxia | 4.44×10^-9^ | 4.30×10^-1^ |
| spastic paraplegia | Tetraplegia | 5.13×10^-15^ |  |
| Amyotrophic lateral sclerosis | Ataxia | 6.94×10^-2^ |  |
| Amyotrophic lateral sclerosis | frontotemporal dementia | 0 | 0 |
| Amyotrophic lateral sclerosis | Myopathies | 2.01×10^-28^ |  |
| Ataxia | Myopathies | 8.97×10^-3^ | 2.78×10^-4^ |
| frontotemporal dementia | Myopathies | 6.71×10^-34^ | 7.80×10^-4^ |
| frontotemporal dementia | Aphasia | 1.92×10^-273^ |  |
| cerebral palsy | Tetraplegia | 5.08×10^-44^ |  |

^a^ Gene P: The binominal test P-value on the number overlapped genes between two diseases;

^b^M_P: The binomial test P-value on the number of overlapped genes between two diseases


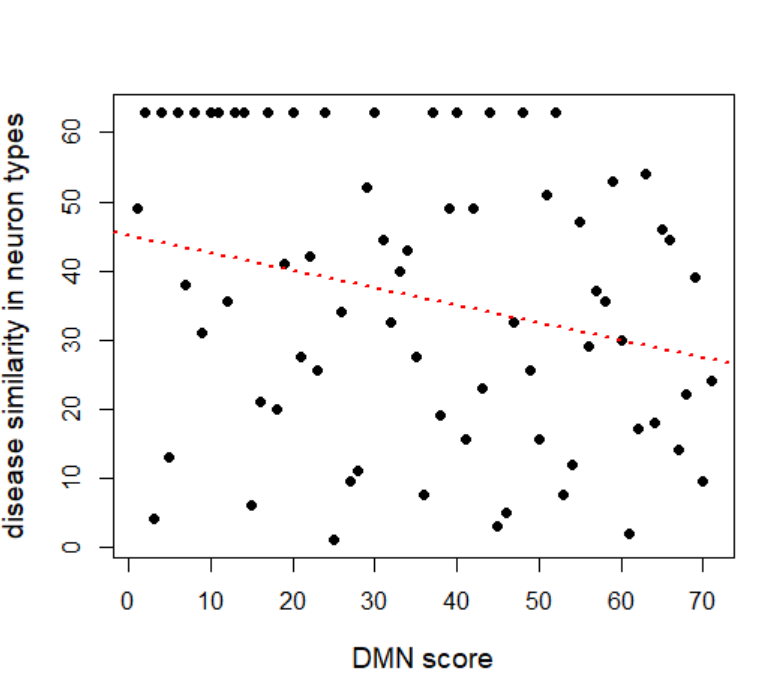


**Rank of enrichment vectors distance**

**Rank of DMN score**

Figure S1. The Spearman correlation coefficient of DMN score and the enrichment profile distance.


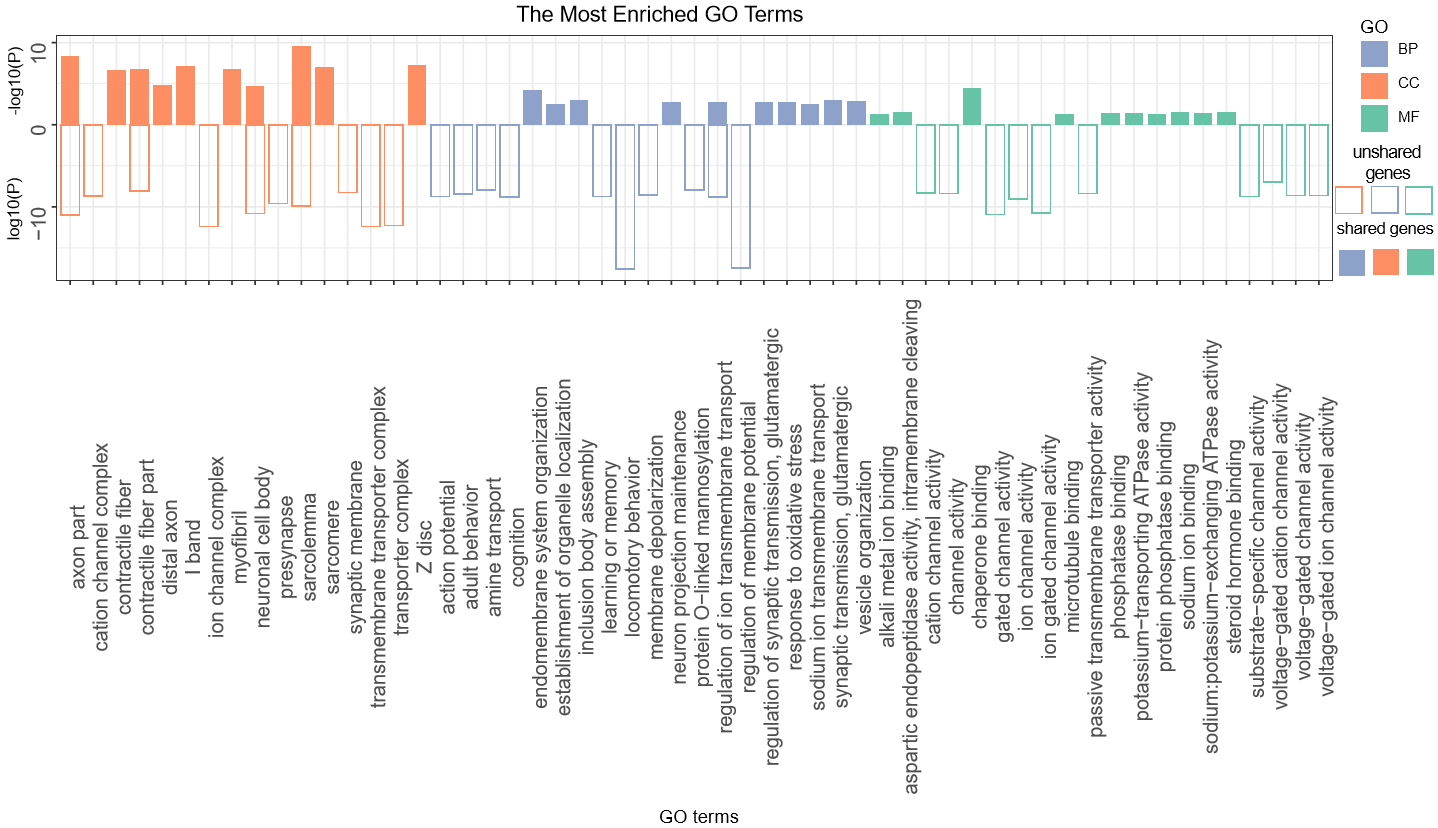


Figure S2. Functional analysis on the shared genes and the unshared genes. The shared genes are involved in multiple functions not involved by any unshared genes.
